# Spectrum Allocation and User Scheduling Based on Combinatorial Multi-Armed Bandit for 5G Massive MIMO

**DOI:** 10.3390/s23177512

**Published:** 2023-08-29

**Authors:** Jian Dou, Xuan Liu, Shuang Qie, Jiayi Li, Chaoliang Wang

**Affiliations:** 1 China Electric Power Research Institute, Beijing 100192, China; liuxuan@epri.sgcc.com.cn (X.L.); qieshuang@epri.sgcc.com.cn (S.Q.); lijiayi@epri.sgcc.com.cn (J.L.); 2 State Grid Zhejiang Electric Power Co., Ltd., Hangzhou 311121, China; wang_chaoliang@zj.sgcc.com.cn

**Keywords:** massive MIMO, spectrum allocation, user scheduling, multi-armed bandit

## Abstract

As a key 5G technology, massive multiple-input multiple-output (MIMO) can effectively improve system capacity and reduce latency. This paper proposes a user scheduling and spectrum allocation method based on combinatorial multi-armed bandit (CMAB) for a massive MIMO system. Compared with traditional methods, the proposed CMAB-based method can avoid channel estimation for all users, significantly reduce pilot overhead, and improve spectral efficiency. Specifically, the proposed method is a two-stage method; in the first stage, we transform the user scheduling problem into a CMAB problem, with each user being referred to as a base arm and the energy of the channel being considered a reward. A linear upper confidence bound (UCB) arm selection algorithm is proposed. It is proved that the proposed user scheduling algorithm experiences logarithmic regret over time. In the second stage, by grouping the statistical channel state information (CSI), such that the statistical CSI of the users in the angular domain in different groups is approximately orthogonal, we are able to select one user in each group and allocate a subcarrier to the selected users, so that the channels of users on each subcarrier are approximately orthogonal, which can reduce the inter-user interference and improve the spectral efficiency. The simulation results validate that the proposed method has a high spectral efficiency.

## 1. Introduction

The development of new applications requires that the data rate be significantly increased. However, a significant increase in data traffic represents a huge challenge to the scarce spectrum resources [1,2]. Massive MIMO has become a key technology in 5G communication system, which greatly improves the system capacity and reduces latency by equipping base stations (BSs) and/or users with a large number of antennas [3,4]. Millimeter wave technology has received significant attention due to its large bandwidth. The combination of millimeter wave and massive MIMO can effectively improve the bandwidth and spectrum utilization of the system [5,6]. On the one hand, the millimeter wavelength is very short, which is conducive to the deployment of a large number of antennas in the system. On the other hand, antenna arrays in massive MIMO systems can bring greater freedom to the system, and using simple linear precoding at the BS can enable the system to achieve a high transmission performance [7,8]. Thus, massive MIMO can compensate for the propagation loss of millimeter wave channels. Therefore, millimeter wave technology is very suitable for massive MIMO systems. In order to fully utilize the gains brought by millimeter wave massive MIMO, it is usually necessary to obtain the channel state information (CSI).

If instantaneous CSI is not available, then non-coherent (NC) techniques are considered key solutions for massive MIMO systems [9,10,11,12]. The designs of NC techniques for massive MIMO systems are mainly focused on energy detection [9,10] or phase detection [11,12]. Manolakos et al. proposed a soft-output detector where the transmitter modulated information during symbol amplitude determination, and the receiver obtained the average received energy [9]. Ngo et al. calculated the marginal posterior of each transmitted signal using the channel distribution and proposed a novel soft-output multi-user detector [10]. Baeza et al. proposed a new constellation design for a multi-user noncoherent large-scale single-input multiple-output uplink system based on *M*-ary differential phase-shift keying [11]. Additionally, non-orthogonal constellation-based schemes to reduce the complexity and increase the spectral efficiency were proposed in [12]. If instantaneous CSI is available, then massive MIMO systems are said to be coherent and have high spectral efficiency due to the available instantaneous CSI [10]. In such scenarios, the symbols are usually from a scalar constellation, such as the quadrature amplitude modulation. In this paper, we consider coherent massive MIMO systems.

The massive MIMO system can serve multiple users on a single time and frequency resource. There have been many studies on the design of transmission schemes for single-carrier massive MIMO systems. Due to the large bandwidth provided by millimeter waves, multiple subcarriers can be utilized to transmit information. How to allocate subcarriers to maximize the spectral efficiency of the system is worth studying. In orthogonal frequency-division multiplexing (OFDM) systems, there are multiple subcarriers to which the spectrum should be allocated. A simple way to achieve this is to randomly allocate the subcarriers to the users. However, this random scheme achieves a relatively low spectral efficiency. Zhang et al. considered a subcarrier allocation method in which the user moves, and each user is allocated one subcarrier [13]. In order to further improve the system’s spectral efficiency, Anand et al. proposed allocating multiple subcarriers to each user to ensure service quality [14]. However, the above studies did not consider the user scheduling problem.

The number of users that a BS can serve in one slot is limited, the system cannot provide services for all candidate users when the number of users is large. Therefore, the user scheduling scheme plays a crucial role in the overall performance of the system [15]. In massive MIMO systems, a simple scheduling scheme is a random scheduling scheme; it randomly selects multiple users for communication. This method does not consider channel information and, therefore, demonstrates poor performance. The authors in [16] proposed a sub-optimal user scheduling algorithm, which performs QR decomposition on the channel and schedules based on the results of QR decomposition, not only reducing the computational complexity but also improving the spectral efficiency. Shehata et al. proposed an iterative method to select terminals in turn by using the eigenvalue and condition number of the channel matrix between the BS and terminals [17]. Bu et al. considered the user scheduling and spectral allocation problem in massive MIMO systems, and proposed a reinforcement learning-based algorithm to improve the spectral efficiency [18]. However, all of these methods require the instantaneous CSI of the terminal. When there are many terminals, it is difficult to obtain the instantaneous CSI of all terminals simultaneously, meaning that these methods are no longer applicable. In addition, due to the slower variation of the statistical CSI compared to instantaneous CSI, user scheduling through statistical CSI can avoid obtaining instantaneous CSI, reduce pilot overhead, and improve spectral efficiency [19]. However, the estimation of statistical CSI also requires a large overhead. When the terminal moves fast, the changes in statistical CSI will also become fast, causing the system performance to deteriorate.

This paper proposes a user scheduling and spectrum allocation scheme based on combinatorial multi-armed bandit (CMAB) for a multi-user millimeter wave massive MIMO OFDM system. The proposed CMAB-based scheme avoids the need for channel estimation for all users, which can reduce pilot overhead and improve spectral efficiency. Our approach utilizes the multi-path channel model in millimeter waves [20]. The proposed scheme is a two-stage scheme. In the first stage, we transform the user scheduling problem into a CMAB problem, and the upper confidence bound (UCB) strategy is used to train and obtain the scheduled users to avoid channel estimation for each user and to improve the spectral efficiency. Each user is regarded as a base arm in the CMAB problem, and multiple users are regarded as the super arm. To improve the system’s spectral efficiency, we aim to maximize the receiving energy. A linear UCB algorithm with low computational complexity is proposed. By setting the UCB value of each base arm, the UCB value of the super arm consists of a linear combination of the UCB value of each base arm, and we select the super arm to maximize the UCB value and the received energy. In addition, it is theoretically proven that the regret of the proposed scheme grows logarithmically over time. In the second stage, the instantaneous CSI estimated in previous slots is used to calculate the statistical CSI, and subcarrier allocation is performed based on the statistical CSI. By grouping the statistical CSI, such that the statistical CSI between different groups is approximately orthogonal, we are able to select one user in each group and allocate one subcarrier to the selected users, resulting in the users in each subcarrier being approximately orthogonal and, thus, improving the spectral efficiency. The simulation results show that the proposed method can significantly improve the spectral efficiency compared with previous methods.

## 2. Preliminaries

### 2.1. System Model

Consider a millimeter wave uplink massive MIMO OFDM system in a single cell with multiple users, where the BS is equipped with a uniform linear array of antennas serving multiple single-antenna users. The communication bandwidth *B* is divided into *F* subcarriers with subcarrier spacing $\frac{B}{F}$. This paper adopts a digital combiner design scheme at the BS [21]. Let $h_{k,f,t}$ be the channel vector between the BS and user *k* in the *t*-th slot at the *f*-th subcarrier and $s_{k,f,t}$ be the transmitted signal from user *k*. Then the received signal at the BS at the *f*-th subcarrier is

(1) $$y_{f,t}=W_{f,t}\sum_{k=1}^{K}h_{k,f,t}^{H}s_{k,f,t}+n_{f,t},$$

where $W_{f,t}\in C^{M\times 1}$ is the digital combiner in the *t*-th slot at the *f*-th subcarrier, and $n_{f,t}$ is the noise. Each element in $n_{f,t}$ follows a Gaussian distribution whose mean is zero and variance is $\sigma^{2}$.

In this paper, we consider a multi-path channel model in millimeter wave communications, and the channel is narrowband time-varying [13]. The channel vector between the BS and user *k* is given by [21]

(2) $$h_{k,f,t}=\sum_{l=1}^{L_{k,f,t}}\rho_{t}\left(\theta_{k,f,l,t}\right)a\left(\theta_{k,f,l,t}\right),$$

where $L_{k,f,t}$ is the number of paths of user *k* at the *f*-th subcarrier, $\rho_{t}\left(\theta_{k,f,l,t}\right)∼CN\left(0,\sigma_{k,f,t}^{2}I\right)$ is the complex path gain, $a\left(\theta_{k,f,l,t}\right)\in C^{M\times 1}$ is the steering vector, and the *i*-th element of $a\left(\theta_{k,f,l,t}\right)$ is $e^{-j2\pi \lambda \frac{i-1}{d}\cos\theta }$. λ is the wavelength, d=λ/2 is the antenna spacing, and $L_{k,f,t}$ is an integer uniformly distributed in $\left\{1,2,\cdots ,L_{max}\right\}$. The power gain $\sigma_{k,f,t}^{2}$ is normalized in $\left[0,1\right]$. Usually, the statistical CSI remains unchanged  for a long time. Suppose that the statistical CSI remains unchanged in *T* slots; this means that the angles $\theta_{k,f,l,t}$ remain unchanged in *T* slots, and the covariance $\sigma_{k,f,t}^{2}$ also remains unchanged. Then, in *T* slots, the channel vector in (2) can be transformed into

(3) $$h_{k,f,t}=\sum_{l=1}^{L_{k,f}}\rho_{t}\left(\theta_{k,l}\right)a\left(\theta_{k,l}\right),$$

where $\rho_{t}\left(\theta_{k,l}\right)∼CN\left(0,\sigma_{k}^{2}I\right)$, and $\sigma_{k}^{2}$ is normalized in $\left[0,1\right]$.

In the user scheduling problem, the conventional schemes design the user scheduling based on the instantaneous CSI. Then, it is necessary for the BS to obtain the instantaneous CSI of all users in real time. Due to the large number of users, it takes a lot of time to estimate the channel, which will lead to a decrease in the system performance. This paper proposes a method based on multi-armed bandit for user scheduling and proposes a sub-carrier allocation method based on statistical CSI. In the process of spectrum allocation and user scheduling, this paper assigns each user a subcarrier and serves *U* users on each subcarrier. Key notations used in this work are listed in Table 1.

### 2.2. The Description of the CMAB

The MAB problem [22] revolves around selecting actions with the goal of maximizing rewards and expectations for the selected actions. After each action is executed, a reward is obtained, and the expectation of the reward is called the value of the action. The value of the action at the *t*-th slot is $Q_{t}\left(A_{i}\right)$, which can be calculated as

(4) $$Q_{t}\left(A_{i}\right)=\frac{\sum_{i=1}^{t-1}R_{i}\cdot 1_{A_{i}=1}}{\sum_{i=1}^{t-1}1_{A_{i}=1}},$$

where $R_{i}$ denotes the reward of the action $A_{i}$ in the *i*-th slot. When the action $A_{i}$ at the *i*-th slot is chosen, then $1_{A_{i}=1}$ is 1, otherwise, $1_{A_{i}=1}$ is 0. Then, it is possible to evaluate the quality $Q_{t}\left(A_{i}\right)$ of actions and develop strategies for selecting actions, including ϵ greedy strategies, UCB strategies, etc. [22]. Unlike the MAB problem, in the CMAB problem [23], multiple actions need to be selected each time, and the reward expectation for multiple actions needs to be maximized. Each action is called a base arm, while the multiple actions selected each time are called a super arm. A super arm consists of multiple base arms.

## 3. Two-Stage Spectrum Allocation and User Scheduling Scheme

### 3.1. The User Scheduling Problem Formulation

In each time slot, we select UF users among *K* users for communication. Each subcarrier serves *U* users, and there are *F* subcarriers in total. The purpose is to enable the selected users to obtain a large spectral efficiency. The spectral efficiency is related to the signal-to-interference-plus-noise ratio (SINR). The received energy is composed of the energy of the received useful signal and the energy of the Gaussian noise. By maximizing the received energy, the energy of the received useful signal will be increased, resulting in improved SINR and spectral efficiency. It should be noted that the received energy is affected by the channel energy. The larger the channel energy, the greater the received energy. Therefore, in order to improve the spectral efficiency of the system, in this paper, we hope that the channel energy of the selected UF users is large. Assuming that the set of selected users in the *t*-th slot at the subcarrier *f* is $A_{f,t}$, let ${\tilde{H}}_{f}\left(t\right)$ be the channel matrix of the selected users in the *t*-th slot at the subcarrier *f*. We define ${∥{\tilde{H}}_{f}\left(t\right)∥}_{F}^{2}$ as the equivalent channel energy of the selected users. The problem of maximizing the channel energy can be formulated as

(5) $$\max\sum_{t=1}^{T}\sum_{f=1}^{F}{∥{\tilde{H}}_{f}\left(t\right)∥}_{F}^{2}$$

In the above problem, due to the large number of users, it is difficult to estimate the instantaneous CSI of all users simultaneously. Without the CSI of all terminals, traditional optimization-based scheduling schemes are not feasible. This paper adopts a method based on a CMAB to select users.

We first transform the user scheduling problem into a CMAB problem. We define *K* users as *K* base arms of the CMAB problem, denoted as 1,2,⋯,K. Each user (base arm) has a channel energy. As shown in Section 2, the CSI of user *k* corresponding to the subcarrier *f* at the *t*-th slot is $h_{k,f,t}$, and let ${∥h_{k,f,t}∥}_{2}^{2}$ be the reward of the base arm *k*. In each time slot, the selected users on all subcarriers are marked as a super arm, which is denoted as $A_{t}$. At this point, the reward for the super arm $A_{t}$ is

(6) $$r_{A_{t}}=\sum_{f=1}^{F}{∥{\tilde{H}}_{f}\left(t\right)∥}_{F}^{2}=\sum_{k\in A_{t}}{∥h_{k,f,t}∥}_{2}^{2}.$$

We select the users in each time slot based on a strategy to maximize the total reward. Assuming that the set of users selected for the *t*-th slot is $A_{t}$, and the set of the users selected on the subcarrier *f* is $A_{f,t}$, we have $A_{t}={⋃}_{f}A_{f,t}$.

When the users scheduled in each subcarrier are obtained, the coherent transmission scheme is shown as follows. Due to the lack of interference between signals on different subcarriers, when estimating the channel, the pilot signal sent by each user on the subcarrier *f* to the base station is $X_{f}$. Therefore, the signal received at the base station on the subcarrier *f* in the *t*-th slot is

(7) $$Y_{f}\left(t\right)={\tilde{H}}_{f}\left(t\right)X_{f}+N_{f}\left(t\right),$$

where $N_{f}\left(t\right)$ is the noise matrix in the *t*-th slot at subcarrier *f*, and each element of $N_{f}\left(t\right)$ is a Gaussian noise with zero mean and the variance is $\sigma^{2}$. Then we use the zero forcing criterion to estimate the instantaneous CSI. By multiplying (7) with $X_{f}^{H}$, we can obtain

(8) $$Y_{f}\left(t\right)X_{f}^{H}={\tilde{H}}_{f}\left(t\right)+N_{f}\left(t\right)X_{f}^{H}.$$

Then the estimation of ${\tilde{H}}_{f}\left(t\right)$ is ${\hat{\tilde{H}}}_{f}\left(t\right)=Y_{f}\left(t\right)X_{f}^{H}$. After the channel estimation is obtained, the MMSE detection operation is adopted for the channels on each subcarrier, so as to reduce the interference and improve the spectral efficiency of the system. When using minimum mean square error (MMSE) detection, the combining matrix $W_{f}\left(t\right)$ is

(9) $$W_{f}\left(t\right)={\left({\hat{\tilde{H}}}_{f}^{H}\left(t\right){\hat{\tilde{H}}}_{f}\left(t\right)+\sigma^{2}I\right)}^{†}{\hat{\tilde{H}}}_{f}^{H}\left(t\right)\left(t\right),$$

where ${\left(\cdot \right)}^{†}$ is the pseudo-inverse operation. The spectral efficiency of the system is

(10) $$R=\sum_{f=1}^{F}\sum_{u=1}^{U}\log_{2}\left(1+\frac{{\left|{\tilde{h}}_{f,u}^{H}\left(t\right)w_{f,u}\left(t\right)\right|}^{2}}{\sum_{l\neq u}{\left|{\tilde{h}}_{f,u}^{H}\left(t\right)w_{f,l}\left(t\right)\right|}^{2}+\sigma^{2}}\right),$$

where $w_{f,u}$ is the *u*-th column of $W_{f}\left(t\right)$, ${\tilde{h}}_{f,u}\left(t\right)$ is the *u*-th column of ${\tilde{H}}_{f}\left(t\right)$, and $\sigma^{2}$ is the variance of the Gaussian noise.

### 3.2. Linear UCB User Scheduling Algorithm

From above, it can be seen that the channels of each user cannot be obtained simultaneously, so traditional optimization methods using the statistical CSI cannot be used. We propose the CMAB method for user scheduling. For the above model, it can be seen that in the multi-path channel model, the reward of user *k* (basic arm *k*) is

(11) $${∥h_{k,f,t}∥}_{2}^{2}=\sum_{l=1}^{L_{k,t}}{\left|\rho_{t}\left(\theta_{k,l}\right)\right|}^{2},$$

where $\rho_{t}\left(\theta_{k,l}\right)∼\mathrm{CN}\left(0,\sigma_{k,l}^{2}\right)$ is a Chi-squared distributed random variable with the degree of freedom 1, and it also follows a sub-exponential distribution with parameter $\left(\sigma_{k,l}^{2},4\sigma_{k,l}\right)$ [24]. According to the characteristics of sub-exponential distribution, $\sum_{l=1}^{L_{k,t}}{\left|\rho_{t}\left(\theta_{k,l}\right)\right|}^{2}$ is also a sub-exponential distribution with parameter $\left(\sum_{l=1}^{L_{k,t}}\sigma_{k,l}^{2},\max_{l}4\sigma_{k,l}\right)$. From Section 2, $\sigma_{k,l}^{2}$ is normalized in [0,1].

Due to the large number of user combinations, the number of super arms grows exponentially with the number of users. In order to reduce the complexity of the algorithm—considering that the UCB strategy can provide a better performance—we propose a linear UCB strategy. This strategy has a complexity that is linear with the total number of users. Since the reward for the super arm is the linear sum of each combination arm, in the linear UCB strategy, the UCB value of the combination arm is set to the sum of the UCB values of the base arm. Considering that the reward of each arm obeys sub-exponential distribution, the UCB value of each arm is defined as

(12) $$u_{i}\left(t\right)=\left\{\begin{array}{cc} {\bar{R}}_{i,t-1}+\bar{c}\max\left\{\frac{16\left(U+1\right)\ln\left(t\right)}{m_{i,t-1},}\right.\left.\sqrt{\frac{2L_{k}\left(U+1\right)\ln\left(t\right)}{m_{i,t-1}}}\right\} & m_{i,t-1}\neq 0,\\ \infty & m_{i,t-1}=0, \end{array}\right.$$

where $\bar{c}=\max\sigma_{k,f,l}^{2}$, $m_{i,t}$ denotes the number of the action *i* that has been selected, and ${\bar{R}}_{i,t}=\frac{{\bar{R}}_{i,t-1}m_{i,t-1}+r_{i,t-1}}{m_{i,t-1}+1}$ denotes the mean reward. From $\sigma_{l,k}^{2}\in \left[0,1\right]$, we have $\bar{c}=1$. Then we define the UCB value of the super arm $A_{t}$ as

(13) $${\bar{u}}_{A_{t}}=\sum_{i\in A_{t}}u_{i}\left(t\right).$$

It can be seen that the UCB values of each super arm can be calculated based on the UCB values of the base arm, greatly reducing the computational complexity. In the *t*-th slot, we select $A_{t}$ to maximize the UCB value of the super arm, and the problem can be described as

(14) $$\max_{A_{t}}{\bar{u}}_{A_{t}}=\max_{A_{t}}\sum_{u\in A_{t}}^{}u_{i}\left(t\right).$$

The above problem is an unconstrained discrete optimization problem. In order to obtain the optimal solution, we calculate the UCB values for all actions, and then sort the UCB value $u_{i}\left(t\right)$. Finally, the users corresponding to the FU-largest UCB values are selected for communication. The user scheduling algorithm is shown in Algorithm 1.
**Algorithm 1:** The CMAB-based user scheduling algorithm
 **Input:** 
$m_{i,t-1}$, ${\bar{R}}_{i,t-1}$;  1:Use (12) to calculate the UCB values of all the base arms;  2:Sort the UCB values of all the base arms;  3:Select the users corresponding to the FU-largest UCB values; the set of these users are $A_{t}$;  4:For each action $a\in A_{t}$, set $m_{a,t}=m_{a,t-1}+1$ and ${\bar{R}}_{a,t}=\frac{{\bar{R}}_{a,t-1}m_{a,t-1}+r_{a,t-1}}{m_{a,t-1}+1}$;
 **Output:** 
$A_{t}$.

**Theorem 1.**  *The regret of the proposed linear UCB scheduling algorithm is upper-bounded by O(lnt).*

**Proof.**  Firstly, we define a variable $B_{i,t}$ to record the number of times the base arm *i* has been selected in the *t*-th slot. Considering the *t*-th slot, if the optimal combination arm $A^{*}$ is selected, the value $B_{i,t}$ will not change. If a non-optimal super arm $A_{t}$ is selected, then $B_{i,t}$ is added by 1 in the *t*-th slot, where $i=\min_{j\in A_{t}}m_{j,t}$. It is easy to prove that in the *t*-th slot, the number of times a non-optimal combination arm is selected is $\sum_{i=1}^{M}B_{i,t}$. Then, we define a variable $V_{i,t}$. If the value $B_{i,t}$ at the *t*-th slot is increased by 1, $V_{i,t}$ is 1. If the value $B_{i,t}$ remains unchanged, then $V_{i,t}$ is set to 0. Let *l* be a positive integer, then we have

(15) $$B_{i,t}=\sum_{p=1}^{t}1\left\{V_{i,p}=1\right\}\leq l+\sum_{p=1}^{t}1\left\{V_{i,p}=1,B_{i,p-1}\geq l\right\},$$

where $1\left\{a\right\}$ is a function. If event *a* is true, then $1\left\{a\right\}=1$. When event *a* is false, then $1\left\{a\right\}=0$. If $V_{i,p}=1$, a non-optimal action is selected, and

(16) $$\begin{array}{ccc} & & B_{i,t}\leq l+\sum_{p=1}^{t}1\left\{\sum_{j\in A^{*}}{\bar{R}}_{j,p-1}+v_{j,p-1}\leq \sum_{j\in A_{p}}{\bar{R}}_{j,p-1}+v_{j,p-1},B_{i,p-1}\geq l\right\}\leq \\ & & l+\sum_{p=1}^{t}1\left\{\sum_{j\in A^{*}}{\bar{R}}_{j,p}+v_{j,p}\leq \sum_{j\in A_{p+1}}{\bar{R}}_{j,p}+v_{j,p},B_{i,p}\geq l\right\}, \end{array}$$

where

(17) $$\begin{array}{c} v_{j,p}=\bar{c}\max\left\{\frac{16\left(M+1\right)\ln\left(p\right)}{m_{j,p}},\sqrt{\frac{2K(M+1)\ln(p)}{m_{j,p}}}\right\}. \end{array}$$

From $\left|A^{*}\right|=U$, $\left|A_{p+1}\right|=U$, and $l\leq B_{i,p}\leq m_{i,p}$, we have

(18) $$\begin{array}{ccc} & & \sum_{p=1}^{t}1\left\{\min_{0<m_{h_{1},t_{1}},m_{h_{2},t_{2}},\cdots ,m_{h_{Q^{*}},t_{Q^{*}}}\leq p}\sum_{j=1}^{Q^{*}}{\bar{R}}_{h_{j},t_{j}}+v_{h_{j},t_{j}}\right.\\ & & \leq \left.\max_{l\leq m_{g_{1},t_{1}},m_{g_{2},t_{2}},\cdots ,m_{g_{Q^{p}},t_{Q^{p}}}\leq p}\sum_{j=1}^{Q^{p}}{\bar{R}}_{g_{j},t_{j}}+v_{g_{j},t_{j}}\right\}\\ & & \leq l+\sum_{p=1}^{\infty }\sum_{m_{h_{1},t_{1}}=1}^{p}\cdots \sum_{m_{h_{Q^{*}},t_{Q^{*}}}=1}^{p}\sum_{m_{g_{1},t_{1}}=l}^{p}\cdots \sum_{m_{g_{Q^{p}},t_{Q^{p}}}=l}^{p}\\ & & 1\left\{\sum_{j=1}^{Q^{*}}{\bar{R}}_{h_{j},t_{j}}+v_{h_{j},t_{j}}\leq \sum_{j=1}^{Q^{p}}{\bar{R}}_{g_{j},t_{j}}+v_{g_{j},t_{j}}\right\}. \end{array}$$

If $\sum_{j=1}^{Q^{*}}{\bar{R}}_{h_{j},t_{j}}+v_{h_{j},t_{j}}\leq \sum_{j=1}^{Q^{p}}{\bar{R}}_{g_{j},t_{j}}+v_{g_{j},t_{j}}$, then one of the following three events must be satisfied.

(19) $$\begin{array}{ccc} & & \varepsilon_{1}:\sum_{j=1}^{Q^{*}}{\bar{R}}_{h_{j},t_{j}}\leq R_{A^{*}}-\sum_{j=1}^{Q^{*}}v_{h_{j},t_{j}}, \end{array}$$

(20) $$\begin{array}{ccc} & & \varepsilon_{2}:\sum_{j=1}^{Q^{p}}{\bar{R}}_{g_{j},t_{j}}\geq R_{A_{p+1}}+\sum_{j=1}^{Q^{p}}v_{g_{j},t_{j}}, \end{array}$$

(21) $$\begin{array}{ccc} & & \varepsilon_{3}:R_{A^{*}}<R_{A_{p+1}}+\sum_{j=1}^{Q^{p}}v_{g_{j},t_{j}}+\sum_{j=1}^{Q^{*}}v_{h_{j},t_{j}}, \end{array}$$

where $R_{A^{*}}=\sum_{j=1}^{Q^{*}}R_{h_{j}},R_{A_{p+1}}=\sum_{j=1}^{Q^{*}}R_{g_{j}}$ and $R_{i}$ is the mean reward of the action *i*. For the event $\varepsilon_{1}$, we have

(22) $$\begin{array}{c} P\left\{E1\right\}\leq \sum_{j=1}^{Q^{*}}P\left\{{\bar{R}}_{h_{j},t_{j}}\leq R_{h_{j}}-v_{h_{j},t_{j}}\right\}. \end{array}$$

Since ${∥h_{k,f,t}∥}_{2}^{2}$ follows a sub-exponential function with parameter $\left(\sum_{l=1}^{L_{k}}\sigma_{k,l}^{2},\max_{l}4\sigma_{k,l}\right)$. Using the Hoeffding inequality, we have

(23) $$\begin{array}{c} P\left\{{\bar{R}}_{h_{j},t_{j}}\leq R_{h_{j}}-c_{h_{j},t_{j}}\right\}\leq 2e^{-\min\left\{\frac{m_{h_{j},t_{j}}c_{h_{j},t_{j}}^{2}}{{\tilde{\tau }}^{2}},\frac{m_{h_{j},t_{j}}c_{h_{j},t_{j}}}{8\bar{\tau }}\right\}}. \end{array}$$

From the above, we have $c_{h_{j},t_{j}}\geq \bar{c}\sqrt{\frac{2K(M+1)\ln t}{m_{h_{j},t_{j}}}}$, and then $e^{-\frac{m_{h_{j},t_{j}}c_{h_{j},t_{j}}^{2}}{{\tilde{\tau }}^{2}}}\leq t^{\frac{-2\left(M+1\right)K{\bar{c}}^{2}}{{\tilde{\tau }}^{2}}}$. Since $K{\bar{c}}^{2}\geq {\tilde{\tau }}^{2}$, we can obtain $e^{-\frac{m_{h_{j},t_{j}}c_{h_{j},t_{j}}^{2}}{{\tilde{\tau }}^{2}}}\leq t^{-2(M+1)}$, $c_{h_{j},t_{j}}\geq \bar{c}\frac{16\left(M+1\right)\ln\left(t-1\right)}{m_{i,t-1}}$ and $e^{-\frac{m_{h_{j},t_{j}}c_{h_{j},t_{j}}}{8\bar{\tau }}}\leq t^{\frac{-2\left(M+1\right)\bar{c}}{\bar{\tau }}}$. Thus we have $e^{-\frac{m_{h_{j},t_{j}}c_{h_{j},t_{j}}^{2}}{{\tilde{\tau }}^{2}}}\leq t^{-2(M+1)}$, and

(24) $$\begin{array}{c} \left(P\left\{E1\right\}\right)\leq Mt^{-2(M+1)}. \end{array}$$

Similarly, we have $\left(P\left\{E2\right\}\right)\leq Mt^{-2(M+1)}$. Let $\Delta_{a\left(t+1\right)}=R_{a^{*}}-R_{a(t+1)}$, and

(25) $$\begin{array}{c} l\geq \max\left\{\left\lceil \frac{32\bar{c}\left(M+1\right)\ln t}{\frac{\Delta_{a\left(t+1\right)}}{M}}\right\rceil ,\left\lceil \frac{8\bar{c}\left(M+1\right)\ln t}{{\left(\frac{\Delta_{a\left(t+1\right)}}{M}\right)}^{2}}\right\rceil \right\}. \end{array}$$

Then we have

(26) $$\begin{array}{c} \sum_{j=1}^{Q^{p}}\frac{16\bar{c}\left(M+1\right)\ln t}{m_{h_{j},t_{j}}}\leq M\frac{16\bar{c}\left(M+1\right)\ln t}{l}\leq M\frac{\bar{c}\left(M+1\right)\ln t}{2\bar{c}\left(M+1\right)\ln t}\frac{\Delta_{a\left(t+1\right)}}{M}=\frac{\Delta_{a\left(t+1\right)}}{2}, \end{array}$$

and

(27) $$\begin{array}{c} \sum_{j=1}^{Q^{p}}\sqrt{\frac{2\bar{c}\left(M+1\right)\ln t}{m_{i,t}}}\leq M\sqrt{\frac{2\bar{c}\left(M+1\right)\ln t}{l}}\leq M\sqrt{\frac{2\bar{c}\left(M+1\right)\Delta_{a\left(t+1\right)}^{2}\ln t}{8\bar{c}\left(M+1\right)M^{2}\ln t}}=\frac{\Delta_{a\left(t+1\right)}}{2}. \end{array}$$

Then we can obtain $\sum_{j=1}^{Q^{p}}c_{g_{j},t_{j}}\leq \frac{\Delta_{a\left(t+1\right)}}{2}$. Meanwhile, we can obtain $\sum_{j=1}^{Q^{*}}c_{h_{j},t_{j}}\leq \frac{\Delta_{a\left(t+1\right)}}{2}$. Thus,

(28) $$\begin{array}{c} R_{a^{*}}-R_{a(p+1)}-\sum_{j=1}^{Q^{p}}c_{h_{j},t_{j}}-\sum_{j=1}^{Q^{*}}c_{h_{j},t_{j}}\geq 0. \end{array}$$

Then, we have $P\left\{E3\right\}=0$. Note that

(29) $$\begin{array}{c} \sum_{p=1}^{\infty }\sum_{m_{h_{1},t_{1}}=1}^{p}\cdots \sum_{m_{h_{Q^{*}},t_{Q^{*}}}=1}^{p}\sum_{m_{g_{1},t_{1}}=l}^{p}\cdots \sum_{m_{g_{Q^{p}},t_{Q^{p}}}=l}^{p}2Mp^{-2\left(M+1\right)}\leq \sum_{p=1}^{\infty }2Mp^{-2}\leq 1+\frac{\pi^{2}}{3}M. \end{array}$$

Thus, $\left(Z_{i,t}\right)\leq l+1+\frac{\pi^{2}}{3}M=O\left(\ln t\right)$, and then

(30) $$\begin{array}{c} Regret\leq \Delta_{max}\sum_{i=1}^{M}\left(Z_{i,t}\right)=O\left(\ln t\right), \end{array}$$

where $\Delta_{max}=max_{a}\Delta_{a}$.    □

### 3.3. Spectrum Allocation Algorithm Based on Statistical CSI Grouping

After obtaining the scheduled users, it is necessary to further allocate the subcarriers to the scheduled users. The goal of subcarrier allocation is to maximize the spectral efficiency. We hope that the user channels on the same subcarrier are approximately orthogonal to each other. If the channels between users are approximately orthogonal, then the inter-user interference will be low, and the spectral efficiency of the users will increase.

Since the instantaneous CSI cannot be obtained, we use the statistical CSI to allocate the subcarriers. Due to the estimation of each user’s channel in the previous slots, the statistical CSI of the users can be calculated based on the instantaneous CSI in the previous slots. Using the statistical CSI to allocate the subcarriers greatly reduces the user’s overhead and improves the spectral efficiency. Suppose the estimation of user *k*’s channel vector in the *t*-th slot at subcarrier *f* is ${\hat{h}}_{k,f,t}$. If this is the case, then its angular domain expression can be obtained as

(31) $$\begin{array}{c} {\hat{h}}_{k,f,t}^{a}=D_{f}{\hat{h}}_{k,f,t} \end{array}$$

where $D_{f}$ is the discrete Fourier transform matrix at the *f*-th subcarrier. This is due to the fact that one user’s channel vector is different on the different subcarriers but the angular domain representation of one user’s channel vector on the different subcarriers are the same. In order to better group the statistical CSI, the calculation method for the statistical CSI on the angular domain (CSIA) is defined as

(32) $$\begin{array}{c} R_{k}^{a}=\frac{1}{t}\sum_{l=1}^{t}{\hat{h}}_{k,f,t}^{a}{\left({\hat{h}}_{k,f,t}^{a}\right)}^{H}. \end{array}$$

After obtaining the CSIA of each user, the next step is to design a subcarrier allocation method. When allocating subcarriers, it is desired that the user channels on each subcarrier are approximately orthogonal. As a result, the serving users are divided into groups, where the channel vectors in different groups are approximately orthogonal. We select one user in each group to obtain one user as the service user on a subcarrier; thus, the users on each subcarrier are more orthogonal, which can lead to higher system spectral efficiency. Considering that the K-means method has good performance in user grouping, this article uses the K-means method to group user statistics into CSI groups. Before using the K-means algorithm, we define a chord distance between X and Y as

(33) $$\begin{array}{c} d_{c}\left(X,Y\right)={∥U_{X}U_{X}^{H}-U_{Y}U_{Y}^{H}∥}_{F}^{2}, \end{array}$$

where $U_{X}$ and $U_{Y}$ denote the eigenvectors corresponding to the non-zero eigenvalues of X and Y, respectively. Then we use an improved K-means algorithm to group the statistical CSI. The difference between the improved K-means algorithm and the K-means algorithm is that the number of users in each group in our algorithm is set to *U*. The details of the spectrum allocation based on the improved K-means grouping algorithm are shown in Algorithm 2.   
**Algorithm 2:** The spectrum allocation based on the improved K-means grouping method
 **Input:** 
$A_{t}$, ${\hat{h}}_{k,f,t^{\prime }},t^{\prime }<t$;  1:Use (31) and (32) to obtain the CSIA for each user;  2:Randomly select *U* users ($u_{1},u_{2},\cdots ,u_{U}$) from the set $A_{t}$;  3:**repeat**  4:    Compute the chordal distance $d_{c}\left(R_{i}^{a},R_{l}^{a}\right)$ between all users with the selected *U* users (i=1,⋯,UF, $l=u_{1},\cdots ,u_{U}$);  5:    **for** l = 1:U **do**  6:        Select F−1 users from the remaining users that are closest to $u_{l}$ and the index set of these users is $S_{l}$;  7:        Calculate the center point $R_{l}^{a}$ of the statistical CSIA of the users in $S_{l}$;  8:    **end for**  9:**until** Convergence  10:**for** f = 1:F **do**  11:    Select one user from each group and allocate these users to the *f*-th subcarrier, the index sets of these users are $A_{f,t},f=1,2,\cdots ,F$;  12:**end for**
 **Output:** 
$A_{f,t}$.

## 4. Simulation Results

We validate the performance of the proposed method through simulation results in this section. Considering the millimeter wave communication scenario, it is assumed that there are a total of *K* mobile users in the cell. The base station is equipped with *M* antennas, and the maximum number $P_{max}$ of paths is set to 4. The number of subcarriers is F=128. The path angles of each user are randomly distributed between $\left[0,180^{\circ }\right]$. The subcarrier spacing is 30 kHz, the number of symbols in each slot is 14, and the slot time is 0.5 ms. Due to the mobile users, the statistical CSI coherence time length is set to 1000 slots (1000 time slots correspond to a range of 5 m at a speed of 10 m/s). After 1000 time slots, the statistical CSI of the users change; that is, the path angle and gain of each user change. We compare the proposed CMAB-based method with statistical CSI-based methods [9], random spectrum allocation, and user scheduling methods. In the method based on statistical CSI, it is assumed that the number of time slots used to estimate statistical CSI is 200, and user scheduling is carried out in the next 800 time slots.

**Figure 1:**
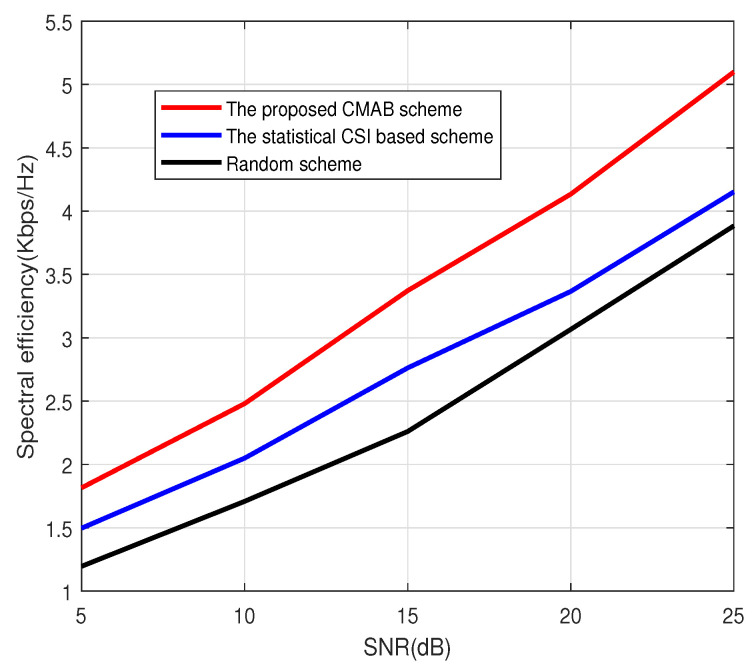
Spectral efficiency vs. SNR. M=64, U=4.

**Figure 2:**
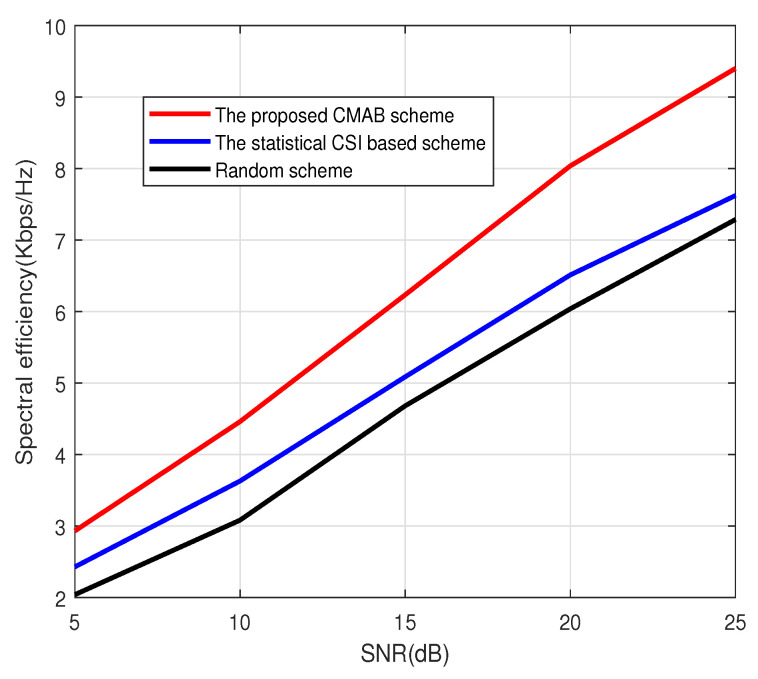
Spectral efficiency vs. SNR. M=64, U=8.

**Figure 3:**
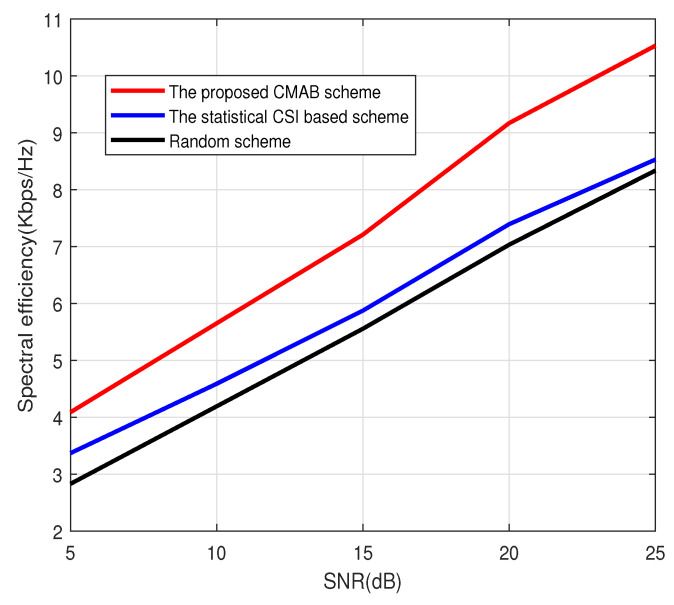
Spectral efficiency vs. SNR. M=128, U=8.

Figure 1, Figure 2 and Figure 3 show the spectral efficiency of the proposed method under different signal-to-noise ratios (SNRs). The number of antennas at BS in Figure 1 and Figure 2 is 64, and the number of antennas at BS in Figure 3 is 128. The total number of users is K=2000. The number of scheduled users in Figure 1 at each subcarrier is 4; that is, the total number of scheduled users in Figure 1 is 512. The number of scheduled users at each subcarrier in Figure 2 and Figure 3 is 8. Figure 1, Figure 2 and Figure 3 show that the CMAB-based method proposed in this paper has a higher spectral efficiency than the statistical CS-based method and the random method. For the scheduling method based on statistical CSI, estimating the channel covariance matrix (CCM) incurs a large pilot overhead, leading to poor average spectral efficiency. The method proposed in this paper does not estimate the statistical CSI and can improve the spectral efficiency. In addition, compared to the random scheduling scheme, the proposed CMAB-based scheme accounts for partial CSI, providing a better channel quality and, thus, improving the spectral efficiency of the system.

**Figure 4:**
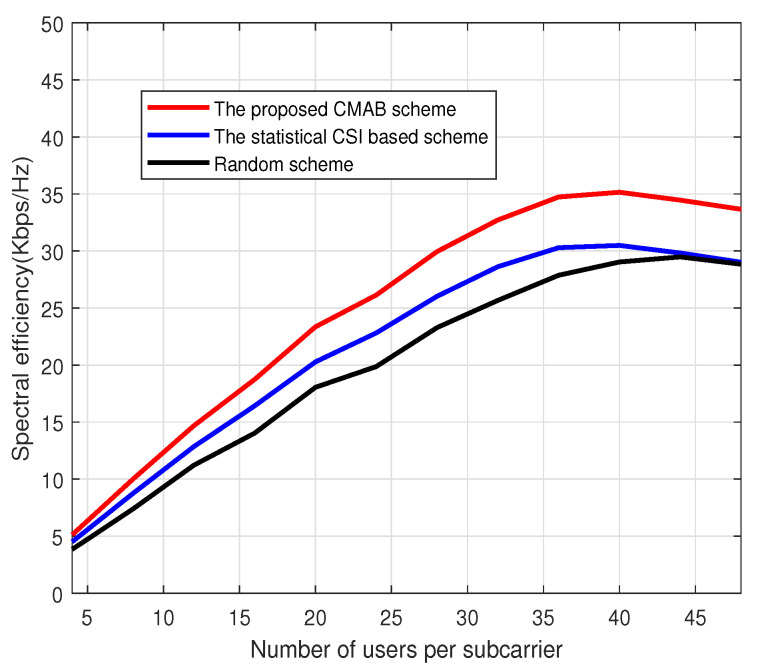
Spectral efficiency vs. users. M=64.

Figure 4 depicts the average spectral efficiency under different numbers of scheduled users per subcarrier. The number of BS antennas is set to 64, and the signal-to-noise ratio is set to 25 dB. The total number of users is 6000. From Figure 4, it can be observed that the method based on CMAB proposed in this paper performs better than the method based on statistical CSI and the random method under a different number of users. In addition, as the number of users increases, the spectral efficiency of all algorithms will increase first and then decrease. When the number of users is small, the inter-user interference is small, and the spectral efficiency will increase as the number of users increases due to user gain. When the number of users is large, the interference between users will increase. Because the number of users is large, user interference will play a major role, and the spectral efficiency of the system will decrease due to the large inter-user interference. Therefore, when the number of users is large, the spectral efficiency of the system will decrease with the increasing number of users.

**Figure 5:**
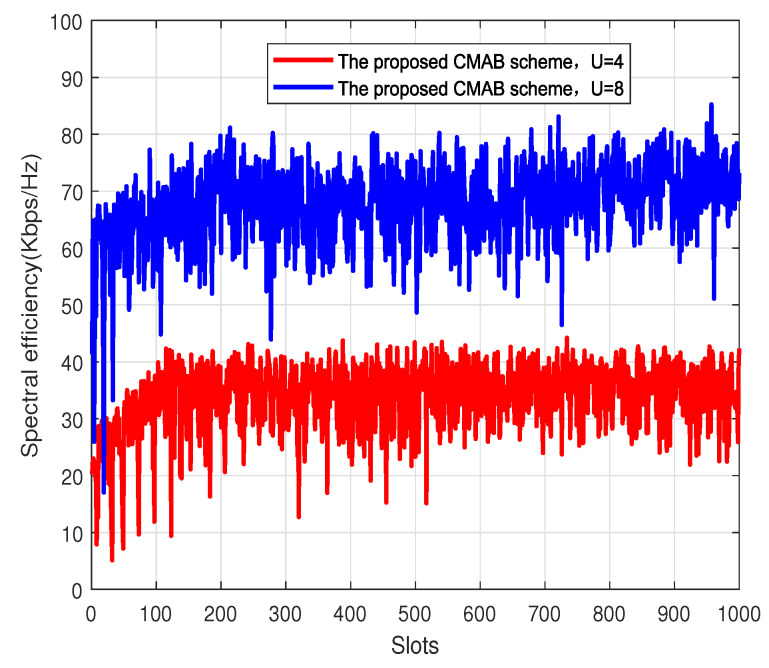
Spectral efficiency vs. slots. M=64.

Figure 5 shows the spectral efficiency of the proposed CMAB-based user scheduling and subcarrier allocation scheme in each time slot. The number of base station antennas is 64, and the signal-to-noise ratio is set to 25 dB. The total number of terminals is 2000, and the user numbers scheduled per subcarrier are four and eight. The spectral efficiency of the proposed CMAB scheme increases first and then converges. The convergence speed when the number of users is four is faster than the convergence speed when the number of users is right. This is because the smaller the number of users, the less time needed to explore. When the number of terminals is large, the spectral efficiency of the system is improved due to user gain, which corresponds to Figure 3; that is, eight users per subcarrier have higher spectral efficiency than four users per subcarrier.

## 5. Conclusions

In this paper, we propose a spectrum allocation and user scheduling scheme based on CMAB for millimeter wave massive MIMO systems. This scheme uses CMAB to schedule the users and uses the statistical CSI to allocate the subcarriers, which can effectively improve the system’s spectral efficiency. Simulation results show that the proposed CMAB-based scheme can effectively improve the spectral efficiency compared with the existing schemes. In future work, we will consider downlink massive MIMO systems. Considering that channel reciprocity does not hold in frequency-division duplex (FDD) massive MIMO systems, a downlink CSI cannot be obtained using the uplink pilot. Thus, designing a transmission scheme for downlink in FDD massive MIMO systems is more challenging. Moreover, it should be noted that a hybrid architecture (including the digital and analogy parts) can reduce hardware complexity; due to this, we will design a user scheduling and spectrum allocation method in a downlink FDD massive MIMO system with hybrid architecture.

## Figures and Tables

**Table 1:** Key notations.

Notations	Parameters
*B*	Bandwidth
*F*	The number of subcarriers
$h_{k,f,t}$	The channel vector between the BS and user *k* in slot *t* at subcarrier *f*
$s_{k,f,t}$	The transmitted signal from user *k* in slot *t* at subcarrier *f*
$W_{f,t}$	The digital combiner in slot *t* at subcarrier *f*
$n_{f,t}$	Gaussian noise vector in slot *t* at subcarrier *f*
$L_{k,f,t}$	The number of paths of user *k* at the *f*-th subcarrier
$\theta_{k,f,l,t}$	The angle of the *l*-th path of user *k* in slot *t* at subcarrier *f*
ρ	The complex path gain
a(·)	The steering vector
λ	the wavelength
*d*	Antenna spacing
$\sigma^{2}$	The variance of the Gaussian noise
$\sigma_{k,f,t}^{2}$	The power gain of user *k* in slot *t* at subcarrier *f*
$\sigma_{k}^{2}$	The power gain of user *k*
$\theta_{k,l}$	The angle of the *l*-th path of user *k* in the *t*-th slot
$A_{i}$	The action
$Q_{t}\left(\cdot \right)$	The value of the action in slot *t*
$R_{i}$	The reward in slot *i*
$A_{f,t}$	The set of selected users in slot *t* at subcarrier *f*
${\tilde{H}}_{f}\left(t\right)$	The channel matrix of the selected users in slot *t* at subcarrier *f*
$A_{t}$	The set of users selected in slot *t*
$X_{f}$	The pilot signal at subcarrier *f*
$Y_{f}$	The received signal at subcarrier *f*
$N_{f}$	The noise matrix in slot *t* at subcarrier *f*
$u_{i}\left(t\right)$	The set of users selected in slot *t*
${\bar{u}}_{A_{t}}$	The UCB value of the super arm $A_{t}$
${\bar{R}}_{i,t}$	The mean reward of action *i* slot *t*
$D_{f}$	The discrete Fourier transform matrix at subcarrier *f*
${\hat{h}}_{k,f,t}^{a}$	The channel vector ${\hat{h}}_{k,f,t}$ in the angular domain
$R_{k}^{a}$	The statistical CSI on the angular domain
$d_{c}\left(X,Y\right)$	The chord distance between X and Y
$U_{X}\left(U_{Y}\right)$	The eigenvectors corresponding to the non-zero eigenvalues of X(**Y**)

## Data Availability

Not applicable.

## Abbreviations

The following abbreviations are used in this manuscript:

MIMO	multiple-input multiple-output
MAB	multi-armed bandit
CMAB	combinatorial multi-armed bandit
UCB	upper confidence bound
CSI	channel state information
BS	base station
NC	non-coherent
TDD	time-division multiplex
OFDM	orthogonal frequency-division multiplexing
MMSE	minimum mean square error
CSIA	channel state information on the angular domain
SNR	signal-to-noise ratio
CCM	channel covariance matrix
FDD	frequency-division duplex
